# P377: Evaluation of the cleaning and disinfection process at a university hospital in Turkey

**DOI:** 10.1186/2047-2994-2-S1-P377

**Published:** 2013-06-20

**Authors:** H Zengin, Y Gelebek, B Cınar, B Cinar, Y Gelebek, H Aytac, H Aytac, Z Bastug, Z Bastug, S Unal, Y Sardan, C Inkaya, S Unal

**Affiliations:** 1 Infection Control Unit, Hacettepe University Adult Hospital, Turkey; 2 Infection Control Unit, Hacettepe University Oncology Hospital, Turkey; 3 Department of İnternal Medicine, Hacettepe University School of Medicine, Turkey; 4 Guven Hospital, Ankara, Turkey

## Introduction

Colonization of resistant bacteria on surfaces in healthcare facilities are an important source for healthcare associated infections. Inappropriate practice of hand hygiene during the care of infected patients may cause environmental contamination and can spread out to another patient. There for, the healthcare setting requires intensive and frequent cleaning with a wide range of products in order to maintain surface hygiene, and infection prevention and control.

## Objectives

In this study, we aimed to revisit, revise and improve standards of practice for hospital cleaning and disinfection procedures developed by our department.

## Methods

A checklist was prepared for written procedures, questions were asked to 38 personnel during day shift. Auditing was performed by infection control nurses in the clinics and intensive care units via observation and responses recorded on the checklist. Data were entered to the computer.

## Results

Our adult hospital has 650 bed capacity with 22 wards and 8 intensive care units. Cleaning services are purchased with contract and controlled by the infection control unit. There are a total of 84 cleaning personnel, 51 on day shift, 20 work in the evening and 12 at night shifts. The mean age was 34 years, 60% were male, 45% primary school graduate and the mean duration of the work in this area was 6 years. Only 21 % of them were doing cleaning from clean areas to the dirty areas, cleaning wipes were not appropriate in 22% and 31% were unaware of appropriate the disinfectant dilution rate. As for the isolated cases, it was observed that 31% of the staff had insufficient knowledge and skills about cleaning and disinfection in this setting.

## Conclusion

Noncompliance to the standards of practice was high although the majority of the staff had over than six years of’ experience, and knew how and where to use the disinfectant. It was also observed that they diluted the disinfectant properly by using any scale/measurement. Thus, we planned an educational program and scheduled regular audits to improve practice.

## Disclosure of interest

None declared

